# Lung Ischemia–Reperfusion Injury in Lung Transplant Surgery: Where Do We Stand?

**DOI:** 10.3390/antiox14111295

**Published:** 2025-10-28

**Authors:** Lawek Berzenji, Jeroen M. H. Hendriks, Stijn E. Verleden, Suresh Krishan Yogeswaran, Wen Wen, Patrick Lauwers, Geert Verleden, Rudi De Paep, Pieter Mertens, Inez Rodrigus, Dirk Adriaensen, Paul Van Schil

**Affiliations:** 1 Department of Thoracic and Vascular Surgery, Antwerp University Hospital, Drie Eikenstraat 655, 2650 Edegem, Belgium; jeroen.hendriks@uza.be (J.M.H.H.); stijn.verleden@uantwerpen.be (S.E.V.); sureshkrishan.yogeswaran@uza.be (S.K.Y.); wen.wen@uza.be (W.W.); patrick.lauwers@uza.be (P.L.); paul.vanschil@uza.be (P.V.S.); 2 Antwerp Surgical Training, Anatomy and Research Centre, University of Antwerp, 2000 Antwerpen, Belgium; 3 Department of Pulmonology, Antwerp University Hospital, 2650 Edegem, Belgium; geert.verleden@uza.be; 4 Intensive Care Unit, Antwerp University Hospital, 2650 Edegem, Belgium; rudi.depaep@uza.be; 5 Department of Anesthesiology, Antwerp University Hospital, 2650 Edegem, Belgium; pieter.mertens@uza.be; 6 Department of Cardiac Surgery, Antwerp University Hospital, 2650 Edegem, Belgium; inez.rodrigus@uza.be; 7 Laboratory of Cell Biology and Histology, University of Antwerp, 2000 Antwerpen, Belgium; dirk.adriaensen@uantwerpen.be

**Keywords:** lung ischemia–reperfusion injury, thoracic surgery, lung transplantation, primary graft dysfunction

## Abstract

Lung ischemia–reperfusion injury (LIRI) remains a major contributor to perioperative morbidity and mortality in thoracic surgery, especially for lung transplantations, where it is one of the principal drivers of primary graft dysfunction (PGD). Although substantial advances have been made in surgical technique, donor management, and perioperative care, LIRI continues to pose a significant clinical challenge. Mechanistically, LIRI reflects a combined pathology of oxidative stress, endothelial and glycocalyx disruption, innate immune activation, mitochondrial dysfunction, and regulated cell death, resulting in loss of alveolar–capillary barrier integrity and gas exchange failure. Current management is phase-specific and multimodal, spanning donor care and preservation, controlled reperfusion and lung-protective ventilation, and pharmacological treatments. Treatment candidates that target oxidative stress and inflammatory cascades (e.g., antioxidants, complement and adenosine pathways, mesenchymal stromal cell products, and dipeptidyl-peptidase-4 inhibition) show promise, yet translation into a clinical scenario remains difficult. Increasing evidence supports endothelial-preserving and mitochondria-sparing strategies, rigorous perioperative bundles, and biomarker-guided trials to move from pathophysiology to practice. Ultimately, addressing LIRI requires an integrated, multidisciplinary approach that spans surgical, anesthetic, and pharmacologic domains, with the goal of improving both early outcomes and long-term graft survival in lung transplant patients.

## 1. Introduction

Ischemia–reperfusion injury (IRI) refers to the detrimental effects on cellular function and homeostasis that occur when blood flow is restored to previously ischemic tissues [1]. Counterintuitively, the return of perfusion can sometimes cause greater tissue damage than the preceding ischemic episode itself, particularly during the reperfusion phase [1,2]. IRI involves a cascade of processes, such as sterile inflammation, increased oxidative stress, endothelial dysfunction, and activation of programmed cell death pathways (apoptosis) [3]. These events can provoke a widespread systemic response, even leading to remote organ injury and multiple organ failure (MOF) as it progresses [4].

Lungs are particularly susceptible to IRI and lung ischemia–reperfusion injury (LIRI) can arise in a variety of clinical settings, including pulmonary transplantation, oncological lung resections, cardiopulmonary bypass (CPB) during cardiac surgery, and even with the use of tourniquets during orthopedic procedures [1,2,5,6,7,8,9]. The key pathophysiological result is the breakdown of the pulmonary endothelial and epithelial barriers, resulting in pulmonary edema, subsequent impaired gas exchange, and an increase in pulmonary vascular resistance, which can result in pulmonary hypertension [10].

The clinical manifestations of LIRI are diverse and often severe. LIRI may present as acute lung injury (ALI) or acute respiratory distress syndrome (ARDS) following major lung resections, or as primary graft dysfunction (PGD) after lung transplantation, all of which are associated with higher morbidity and mortality rates [11,12]. In lung transplantation procedures, the donor lungs are kept in an ischemic state until placement on ex vivo lung perfusion (EVLP) or implantation, at which point reperfusion in the recipient can trigger LIRI and PGD, frequently resulting in prolonged mechanical ventilation and a longer hospital length of stay [10,12,13]. Furthermore, IRI is the most predominant risk factor for late graft failure in lung transplant procedures, including the development of chronic lung allograft dysfunction (CLAD) [10,14]. Due to the profound impact of LIRI on post-surgical and post-transplant results, significant research has focused on its prevention and management [15,16].

A variety of experimental and preclinical studies have explored pharmacological and non-pharmacological interventions aimed at limiting LIRI. These include the use of specialized organ preservation solutions, optimizing storage temperatures, EVLP, lung-protective ventilation strategies, antioxidants, anti-inflammatory drugs, and stem cell therapies [17,18,19,20,21]. More recently, scientific attention has shifted toward molecular targets, such as the endothelial glycocalyx, mitochondrial processes, and the regulation of autophagy [22,23,24,25]. Among the pharmacological approaches, dipeptidyl peptidase 4 inhibitors (DPP-4i) have emerged as promising agents due to their antioxidant, anti-inflammatory, and anti-apoptotic effects, and their potential to mitigate both acute IRI and chronic allograft injury in experimental models [26,27].

Nevertheless, the complex nature of LIRI pathogenesis and treatment remains a significant challenge. Many proposed interventions remain in the early stages of research or have not yet demonstrated clear benefit in clinical trials. Continued investigation is necessary to unravel the underlying mechanisms of LIRI and to identify and develop effective interventions to improve surgical and transplant outcomes.

In this review, we summarize the most recent advances in the understanding and management of LIRI and highlight novel therapeutic avenues within the broader context of oxidative stress and antioxidant defense.

## 2. Pathophysiology of LIRI

The pathophysiology of LIRI is multifactorial and initiated during an ischemic event or period (e.g., the ischemic storage of donor lungs), when cessation of blood flow leads to rapid depletion of adenosine triphosphate (ATP) and a shift to anaerobic metabolism. This aerobic energy depletion and shift is accompanied by intracellular acidosis, lactate accumulation, and the buildup of purine metabolites such as hypoxanthine, which serve as precursors for the subsequent generation of reactive oxygen species (ROS) by endothelial cells, macrophages, and other immune cells upon reperfusion [4,10,17,28].

Following the depletion of ATP, pulmonary cellular ion homeostasis is rapidly disrupted as ATP-dependent pumps such as the Na^+^/K^+^-ATPase fail, resulting in cellular edema and calcium overload. These changes activate phospholipases and proteases, and initiate the breakdown of cellular and mitochondrial membranes [2,29]. When alveolar oxygen is limited (e.g., during cold storage without ventilation), mitochondrial electron transport slows and succinate accumulates via reversal of succinate dehydrogenase; upon reperfusion, rapid succinate oxidation drives reverse electron transport at complex I, generating a burst of mitochondrial superoxide and amplifying tissue injury. In parallel, and importantly in lungs even when parenchymal oxygenation is preserved, ischemia alters endothelial shear sensing: stop-of-flow is detected by a caveolae-resident “mechanosome” (PECAM-1/VEGFR2/VE-cadherin), leading to KATP-channel closure, endothelial depolarization, opening of T-type Ca^2+^ channels and eNOS activation, and phosphoinositide 3-kinase (PI3K)–Akt–dependent assembly of NOX2 with ROS generation; the abrupt change in shear on reperfusion can further activate NOX2. Notably, because lung parenchyma can remain oxygenated during ischemia if ventilated, NOX2-driven mechanosignaling provides a hypoxia-independent route to ROS in lung IRI, whereas hypoxic storage adds the succinate-mediated mitochondrial ROS burst on reperfusion [3,10,30,31,32].

Reperfusion, although necessary to restore oxygen delivery and limit irreversible tissue loss due to ischemia, paradoxically triggers a dramatic burst of ROS generation [2,4]. This process is driven by both the metabolism of accumulated hypoxanthine by xanthine oxidase and activation of nicotinamide adenine dinucleotide phosphate (NADPH) oxidase isoforms in pulmonary endothelial cells, alveolar macrophages, and infiltrating leukocytes [33,34]. The resultant oxidative stress overwhelms the lung’s endogenous antioxidant defenses, causing the peroxidation of membrane lipids, carbonylation and nitration of proteins, and deoxyribonucleic acid (DNA) strand breaks [35]. This primary oxidative injury is amplified by downstream effects on intracellular signaling pathways that further compromise cellular survival and function [28].

In addition to direct tissue damage, ROS can also serve as potent pro-inflammatory mediators, rapidly activating resident alveolar macrophages and endothelial cells. These cells release a broad array of cytokines and chemokines, including tumor necrosis factor-alpha (TNF-α), interleukin (IL)-1β, CXCL-8, and CXCL2, which initiate a cascade that recruits and activates neutrophils and other innate immune effectors. Neutrophil and macrophage infiltration are prominent features of LIRI and are associated with the release of additional ROS, proteases, elastases, and the formation of neutrophil extracellular traps (NETs), all of which contribute to the destruction of lung parenchyma and the microvascular barrier [3,28,36].

The pulmonary endothelium is a principal target of this injury. Oxidative and inflammatory damage cause cytoskeletal remodeling and disruption of intercellular junctions, resulting in increased vascular permeability and the leakage of protein-rich fluid into the interstitial and alveolar spaces [37,38]. This process is further exacerbated by the activation of the complement and coagulation cascades. Ischemia and early reperfusion trigger all three complement pathways in the lung: natural IgM/neoepitope binding (classical), mannose-binding lectin (MBL) sensing ischemia-induced glycan changes (lectin), and amplification via the alternative pathway. This culminates in generation of C3a/C5a (potent chemoattractants that up-regulate endothelial adhesion, prime neutrophils, and increase permeability) and assembly of the terminal complement complex (C5b-9) that directly injures endothelium and worsens microvascular leak and thrombosis. Clinically, local complement activation in the allograft within 24 h after transplant, i.e., elevated sC4d and sC5b-9 in bronchoalveolar lavage (BAL) and higher levels of C1q/C2/C4 (classical/lectin) and Ba (alternative), is associated with PGD, with MBL and ficolin-3 strongly correlating with terminal complement activity in BAL [39,40]. Systemically, rising plasma C5a between 6–24 h post-reperfusion correlates with both PGD and mortality, supporting a role for an early complement surge [40]. Early C3d/C4d deposition in lung allografts has likewise been observed after transplantation [41]. Together, these data support complement as a key amplifier of LIRI that links oxidative/endothelial injury to neutrophil recruitment, coagulation crosstalk, and early edema.

**Figure 1:**
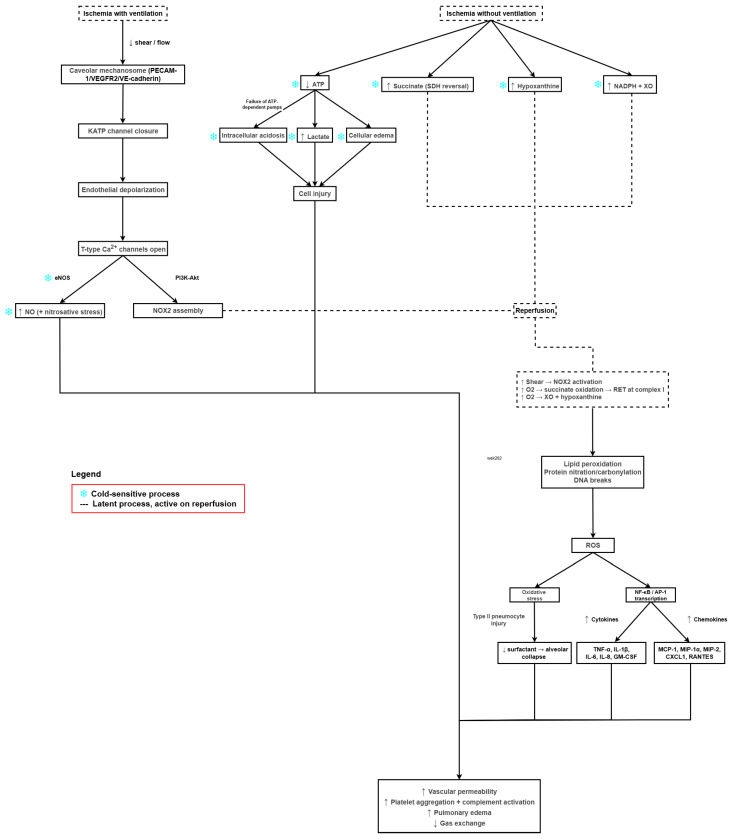
Schematic overview of lung ischemia–reperfusion injury. Ischemia with ventilation (left; mechanotransduction) and without ventilation (middle; oxygen debt) are shown with their respective pathways and links to reperfusion (right). Dashed boxes/lines indicate latent processes expressed on reperfusion; ❄ marks cold-sensitive processes. Reperfusion combines start-of-flow–induced NOX2 activation with O_2_-dependent bursts from succinate oxidation/reverse electron transport (RET) at complex I and from xanthine oxidase (XO). Reactive oxygen species (ROS) drive lipid peroxidation, protein nitration/carbonylation, and DNA breaks and activate NF-κB/AP-1, leading to cytokine/chemokine release, type II pneumocyte injury with ↓surfactant and alveolar collapse, increased vascular permeability, platelet/complement activation, pulmonary edema, and impaired gas exchange. AP-1, Activator Protein-1; ATP, adenosine triphosphate; CXCL1, CXC ligand 1; eNOS, endothelial nitric oxide synthase, GM-CSF, Granulocyte-macrophage colony-stimulating factor; IL, interleukin; KATP, ATP-sensitive potassium channel; MCP-1, Monocyte chemoattractant protein-1; MIP, Macrophage inflammatory protein; NADPH, nicotinamide adenine dinucleotide phosphate; NF-κB, nuclear factor kappa-B; NO, nitric oxide; NOX2, NADPH oxidase-2; PECAM-1, platelet endothelial cell adhesion molecule-1; PI3K–Akt, Phosphoinositide 3-kinase → Akt pathway; RANTES, “Regulated on Activation, Normal T-cell Expressed and Secreted”; ROS, reactive oxygen species; SDH, succinate dehydrogenase; TNF, tumor necrosis factor; VE-cadherin, vascular endothelial cadherin; VEGFR2, vascular endothelial growth factor receptor-2; XO, xanthine oxidase.

In addition, vasoactive mediators such as thromboxane A2 and platelet-activating factor further promote pulmonary edema formation [10]. The alveolar epithelium is also highly vulnerable, particularly its type II pneumocytes; injury to these cells impairs lung surfactant synthesis and turnover, contributing to alveolar instability and collapse, resulting in worsening gas exchange [42]. Figure 1 shows a schematic overview of the pathophysiology of LIRI.

A growing body of evidence has identified the endothelial glycocalyx as a critical modulator of vascular homeostasis in the process of IRI [24,43]. In blood vessels, the endothelial glycocalyx is a glycoprotein- and proteoglycan-rich layer projecting into the vascular lumen that regulates vascular permeability, mechanotransduction, and inflammatory responses [44,45]. Ischemia and ROS-mediated degradation of the glycocalyx enhances leukocyte adhesion, disrupts vascular barrier integrity, and amplifies edema and inflammation [10]. In parallel, mitochondrial dysfunction, driven by calcium overload and sustained oxidative stress, triggers the opening of mitochondrial permeability transition pores and the release of pro-apoptotic factors, resulting in both apoptotic and necrotic cell death [46]. The subsequent release of damage-associated molecular patterns (DAMPs) into the extracellular space perpetuates the cycle of sterile inflammation and tissue injury [28].

While LIRI is initiated within the transplanted lung, the consequences are often systemic. The spillover of cytokines, DAMPs, and activated immune cells into the circulation can precipitate remote organ dysfunction and even failure. Notably, unilateral LIRI can elicit contralateral lung injury [10,28]. Despite advances in lung transplant surgery such as donor management, graft preservation, and surgical technique, these complex, destructive processes remain a significant challenge, and underscore the urgent need for targeted interventions. Novel therapeutic approaches focused on antioxidant administration, immune modulation, and preservation of endothelial integrity hold promise for attenuating LIRI and improving clinical outcomes in major lung surgery and transplantation [25,47].

## 3. Clinical Impact of LIRI–Lung Transplantation

The primary effect of LIRI is the breakdown of the alveolar–capillary barrier, leading to non-cardiogenic pulmonary edema and impaired ventilation-perfusion (V/Q) matching. This disruption causes pulmonary edema, impaired gas exchange, reduced static and dynamic lung compliance, and raises the alveolar-arterial oxygen gradient [(A-a)DO_2_] [1,24]. Reperfusion also triggers precapillary vasoconstriction, which can triple pulmonary vascular resistance, often resulting in pulmonary hypertension and worsening hydrostatic edema. Clinically, severe LIRI presents as ALI or ARDS, with bilateral pulmonary edema and refractory hypoxemia that are indistinguishable from other causes of ARDS [4,48]. There are no specific diagnostic markers, making LIRI essentially a diagnosis of exclusion once cardiogenic edema and other etiologies are ruled out.

### 3.1. Lung Transplantation and PGD

In the context of lung transplantation, LIRI manifests as PGD, an ALI syndrome and the leading cause of early post-transplant morbidity and mortality [3]. It is associated with a 30-day mortality rate that is approximately sevenfold higher than that in patients without PGD [49]. PGD is characterized by hypoxemia (low PaO_2_/FiO_2_ ratio) and diffuse pulmonary infiltrates on chest X-ray without other identifiable causes [50]. Multiple risk factors have been identified; including donor, recipient, and procedural variables. Notable variables include donor smoking and alcohol history, donor age, pulmonary embolism, size mismatch, recipient disease etiology, recipient body mass index (BMI), use of extracorporeal life support perioperatively, ischemic times, polytransfusion, and timing of transplants [15,51]. Many of these risk factors influence the development of PGD through mechanisms that converge on IRI, the central pathophysiologic process underlying early allograft dysfunction. Donor exposures such as smoking and alcohol use are associated with endothelial dysfunction, oxidative stress, and impaired alveolar fluid clearance, which prime the graft for exaggerated inflammatory and oxidative responses upon reperfusion [52,53]. Older donor age similarly heightens susceptibility to IRI owing to reduced cellular stress-response capacity and augmented baseline inflammation [54]. Hemodynamic and mechanical stressors, such as pulmonary hypertension, size mismatch, and obesity, exacerbate microvascular shear and capillary leak during reperfusion, further amplifying IRI-related endothelial injury [55,56,57,58]. Procedural factors including prolonged ischemic times, the use of extracorporeal life support, and excessive transfusion burden can each potentiate the release of DAMPs, complement activation, and neutrophil recruitment, thereby intensifying the inflammatory cascade characteristic of IRI and PGD [10,59,60,61]. These variables likely act in concert rather than isolation, with cumulative “hits” to the donor lung and recipient environment synergistically amplifying the IR response and translating into higher grades of PGD [24].

Histologically, PGD corresponds to diffuse alveolar damage (similar to ARDS) caused largely by IRI to the transplanted lung. LIRI-induced sterile inflammation and damage to transplanted lungs results in diffuse alveolar damage with flooding of alveoli, loss of ventilating surface, and severe gas exchange impairment. Clinically, this manifests as PGD grades 1–3 (with grade 3 being PaO_2_/FiO_2_ < 200 and bilateral infiltrates) in the hours after transplant [15,50]. Severe PGD is not only a leading cause of early post-transplant morbidity and mortality, but is also associated with worse long-term outcomes, including a higher risk of developing CLAD [12]. Despite advances in surgical technique and preservation, LIRI-induced PGD still occurs in up to 30% of lung transplant recipients, underscoring the need to understand its pathophysiology and develop targeted interventions [12,62,63].

The primary approach of PGD prevention lies in identifying and minimizing modifiable risk factors in both donors and recipients, which highlights the necessity of a thorough risk-factor assessment before transplantation [64,65]. Contemporary efforts also focus on optimizing reperfusion and ventilation strategies, along with improving lung preservation techniques, including EVLP, to mitigate LIRI [63]. Extended ischemic (cold storage) time has been consistently recognized as a strong procedure-related risk factor for PGD, underscoring the importance of reducing ischemic duration through careful surgical and anesthetic planning [66]. Preventive strategies therefore include optimizing donor lung storage temperatures, adopting controlled reperfusion protocols, using lung-protective ventilation, and applying targeted pharmacologic interventions to modulate the inflammatory and oxidative pathways that drive LIRI [10,21].

### 3.2. Risk Factors and Selection of Donor/Recipient

Due to the fact that PGD is mainly a clinical expression of LIRI, donor and recipient risk factors are best understood by how they prime the endothelium/epithelium for reperfusion or increase the IRI “dose.” Donor lungs subjected to pre-procurement hypoxemia or hypotension frequently accompany brain death-related catecholamine surges and inflammatory activation, which injure the alveolar–capillary barrier and glycocalyx and lower the threshold for edema and hypoxemia upon reperfusion [10,24,62,67]. Previous studies have shown that these donor lungs with prolonged hypoxemia or hypotension prior to organ retrieval also have higher PGD rates [12,62]. Brain death itself triggers a systemic cascade of cytokines and complement with neutrophil recruitment in the lung; this “first hit” potentiates IRI during implantation, thus priming the lung for PGD after transplant [62,63,67,68,69].

Donor demographics (e.g., increased age) can further heighten IRI susceptibility through reduced stress-response capacity and “inflammaging,” although results vary across cohorts [10,62,67]. Donor smoking and chronic alcohol abuse are consistently associated with higher PGD risk and mortality, possibly via baseline endothelial dysfunction, oxidant burden, impaired surfactant and alveolar fluid clearance, all of which amplify IRI signaling at reperfusion [52,53]. Donor smoking history has been identified as one of the strongest predictors of PGD [52]. Other donor injuries (aspiration, chest trauma) and prolonged mechanical ventilation add inflammatory and ventilator-induced stresses that sensitize the graft to IRI; conversely, donor lung-protective ventilation and careful fluid/hemodynamic management can mitigate these effects [12,50,62,70].

On the recipient side, factors that raise mechanical/hemodynamic stress at first reperfusion or increase baseline inflammatory tone intensify IRI and PGD risk. Higher BMI is associated with a dose–response increase in PGD, likely reflecting systemic low-grade inflammation, impaired respiratory mechanics, and higher peri-operative transfusion/support needs that magnify IRI [71]. Overweight and obese lung transplant recipients have a significantly higher incidence of PGD with an estimated risk increase of 6% for BMI 25–30 and 11% for BMI > 30 [12,55]. Pre-existing pulmonary hypertension in the recipient is another known risk factor, as high pulmonary artery pressures (PAPs) can exacerbate reperfusion injury in the new lung by increasing shear and transvascular hydrostatic load across a vulnerable endothelial barrier during reperfusion [56,72]. Certain diagnoses leading to transplant, such as idiopathic pulmonary fibrosis or sarcoidosis, have been associated with higher PGD rates, possibly due to the severe lung fibrosis/vascular remodeling and inflammation present in those diseases, which can lead to prolonged ischemic exposures and increase initial reperfusion pressures [12,50,56,57,62]. In addition, donor–recipient size matching is an important aspect in modulating LIRI and PGD [73]. Previous studies have shown that oversizing allografts have been linked to a reduced risk of postoperative PGD, particularly in patients without chronic obstructive pulmonary disease (COPD), possibly due to decreased shear upon reperfusion [74]. In summary, carefully optimizing the recipient’s condition and selection of appropriate donor–recipient matches are important steps to reduce LIRI burden and PGD risk.

### 3.3. Donor Procedure and Preservation

The prevalence of IRI-induced PGD and donor shortage in lung transplantation surpasses that of all other organ transplants [10,75]. Therefore, the process of donor lung procurement and preservation needs to minimize ischemic injury and optimally prepare the organ for transplantation.

After flushing, donor lungs are transported in a cooled state to minimize metabolic activity leading to IRI and preserve tissue integrity. Traditional ice-on-cooler methods produce uneven, sometimes near-freezing temperatures around 0 °C, risking cold-induced cellular and endothelial injury and variable cooling across lobes; controlled hypothermia avoids this pitfall [21,76]. However, emerging clinical and translational evidence now supports controlled hypothermic storage at approximately 10 °C as optimal for lung preservation [77,78]. This slightly warmer temperature better maintains mitochondrial function and cellular metabolism, thereby attenuating the metabolic drivers of IRI while avoiding the risks of uneven or excessively cold exposure seen with traditional ice storage [21,77,79,80,81,82,83]. Mechanistically, holding lungs at ~10 °C maintains a low but viable metabolic state that limits ATP collapse and DAMP release, curbs mitochondrial priming (e.g., succinate-driven ROS burst) and protects the endothelium/glycocalyx, all of which translate into a smaller reperfusion inflammatory surge [10,24,81,82,83,84,85]. In a prospective, multicenter, nonrandomized trial, prolonged preservation at 10 °C resulted in low rates of PGD grade 3 and similar outcomes to lungs transplanted using conventional methods with shorter preservation times [86]. Although longer cold storage would ordinarily be expected to increase the ischemic dose, controlled hypothermia at ~10 °C slows metabolism while preserving mitochondrial homeostasis and limiting ischemic succinate build-up, the key driver of the early reperfusion ROS burst, thereby attenuating IRI at reperfusion [21,77,81,86,87]. Consistent with this mechanism, a multicenter clinical series reported safe preservation approaching 24 h without excess PGD [77]. Currently, there is an ongoing randomized, controlled trial investigating whether lung preservation at 10 °C is non-inferior to standard cooling temperatures.

Additionally, the lungs are not completely collapsed during storage; they are partially inflated to about 50% of total lung capacity using a low volume of air or oxygen. This strategy preserves surfactant function and maintains alveolar recruitment, improving compliance and reducing atelectatic injury during the ischemic period [12,88]. Furthermore, maintaining partial inflation during cold storage may limit atelectrauma and necrosis that drive sterile DAMP release, particularly high mobility group box 1 protein (HMGB1), which is implicated in early ischemia–reperfusion lung injury and primary graft dysfunction [89]. The combination of precise hypothermia and controlled partial inflation has been shown to enhance cell viability and membrane integrity, supporting better early graft function following implantation [78].

Both cold and warm ischemia durations are kept as short as possible to prevent LIRI. Prolonged total ischemic time (from donor cross-clamp to reperfusion in the recipient) is a well-established risk factor for IRI-induced PGD [12,90]. In practice, teams coordinate logistics (donor timing, transport, operating room readiness) and increasingly use controlled hypothermia at ~10 °C (with or without EVLP when indicated) to shorten warm phases and buffer the cold phase metabolically, thereby dampening the reperfusion inflammatory cascade and lowering PGD incidence [20,21,77,80,91,92,93].

### 3.4. Lung Implantation Procedure

The intraoperative and immediate postoperative management of a lung transplant are crucial in modulating the severity of LIRI. Perioperatively, the unclamping of the pulmonary artery is generally performed in a stepwise manner, allowing a controlled reperfusion and thus preventing an onslaught of full cardiac output to the cold ischemic lung [94]. By partially unclamping the pulmonary artery or using a brief, low-pressure, low-flow reintroduction of blood (over ~2–5 min, with normoxic FiO_2_), the initial surge in shear stress and oxygen delivery is blunted, allowing endothelial Ca^2+^ handling, nitric-oxide–mediated vasodilation, and glycocalyx recovery, thereby limiting capillary leak and the early mitochondrial ROS burst that drives lung IRI [10,24,84,85,87,88]. This graded reperfusion/postconditioning approach is mechanistically aligned with lung IRI biology: a sudden full-flow, hyperoxic reperfusion increases endothelial shear, glycocalyx shedding, Ca^2+^ overload, and reverse-electron transport (RET)–mediated ROS at complex I fueled by ischemic succinate accumulation. Slowing the first minutes of reperfusion reduces these triggers [10,24,84,85,87]. In lung transplantation, clinical “modified/controlled reperfusion” strategies have been associated with lower severe PGD and better early physiology, and preclinical/EVLP models show that lower initial flow reduces edema and inflammatory cytokines [50,62,95]. Avoiding hyperoxia at first reperfusion (using lower FiO_2_) further attenuates oxidative injury and has been linked to a lower risk of severe PGD [96]. Cross-organ data support the same principle: in the liver, end-ischemic hypothermic oxygenated perfusion (HOPE), often followed by controlled oxygenated rewarming (COR) and then warm normothermic machine perfusion (NMP), metabolizes ischemic succinate and replenishes ATP under low metabolic demand, thereby dampening the subsequent reperfusion burst. Randomized trials in DCD livers show reduced biliary complications with (dual) HOPE versus static cold storage [97,98,99,100,101]. In the heart, stepwise/controlled reflow and ischemic postconditioning reduce infarct size and endothelial dysfunction, reinforcing the value of gradual re-reperfusion to mitigate IRI [102]. Together, these data justify stepwise, normoxic, low-pressure reperfusion of the lung allograft as a practical, mechanism-based countermeasure to LIRI.

Another approach is modified reperfusion, where a catheter is placed in the pulmonary artery in order to administer leukocyte-depleted blood mixed with vasodilators before full circulation is restored. In a series of 100 patients managed with this technique, the incidence of severe PGD was remarkably low (around 2%) and early outcomes were excellent [50,103]. The rationale is that flushing out activated donor leukocytes and providing a vasodilated, controlled flow reduces the “trigger” for endothelial injury at first contact with recipient blood. While not all centers use specialized reperfusion circuits, the general principle is to avoid an immediate high-pressure, high-oxygen surge to the graft [94]. Additionally, reperfusion is ideally done with the lung already being gently ventilated with oxygen to avoid blood flowing into a completely collapsed and hypoxic lung. Some protocols also recommend keeping the FiO_2_ moderate during the first few minutes of reperfusion [62,104]. In fact, use of very high FiO_2_ (>0.4) during initial reperfusion has been associated with higher rates of PGD, likely because excessive oxygen tension exacerbates ROS generation in the newly perfused lung [12,105]. Therefore, many centers will start reperfusion on 40–50% oxygen rather than 100% oxygen, increasing it later as needed [10,16].

Post-implantation, the donor lung is extremely vulnerable to ventilator-induced injury. Mechanical ventilation itself can cause shear stress and inflammatory injury, especially in an acutely injured lung. To avoid compounding the reperfusion injury, a lung-protective ventilation strategy is used from the time of reperfusion onward [96]. This includes low tidal volumes (typically 6–8 mL/kg or less of predicted body weight) and adequate positive end-expiratory pressure (PEEP) to prevent alveolar collapse, similar to ARDS ventilation protocols [12,106]. Studies have shown that tidal volumes > 8–10 mL/kg in the early postoperative period can induce PGD, whereas limiting volumes can decrease neutrophil infiltration and cytokine release in the graft [12]. It is also important to avoid excessive driving pressures; keeping plateau pressures in a safe range (<30 cm H_2_O) and using gentle recruitment maneuvers if needed [96,106]. If a size mismatch occurs (e.g., smaller donor lungs in a larger recipient), special care must be taken to avoid over-ventilating the graft as size mismatches can lead to inadvertent delivery of too large a volume per lung volume [107]. In fact, donor–recipient lung size mismatch is recognized as a modifiable risk factor for PGD, precisely because of the ventilation implications [12,70]. Early extubation can be considered if the patient is stable, as this allows the patient to breathe spontaneously and may reduce ventilator-associated injury; however, this must be balanced against the need for adequate support if the lung is still recovering [108,109].

Transplanted lungs should be protected from acute hydrostatic pressure overload. Reperfusion injury can be worsened by high pulmonary capillary pressures, leading to stress edema [110]. Thus, transplant teams often aim to keep the patient “dry” and limit intravenous (IV) fluids in the intraoperative and immediate postoperative phase [111,112]. In practice, a low central venous pressure (CVP) or PAP is maintained to reduce the burden on the fragile pulmonary capillaries. Inotropic support and vasodilators may be used to optimize the right heart function and pulmonary circulation without volume loading [110]. Some centers avoid CPB for lung transplant when possible, opting for off-pump or ECMO support if needed, as CPB is associated with greater inflammatory response and capillary leak, which can elevate PGD risk [12,62]. If CPB or ECMO is used perioperatively, for example, in cases of hemodynamic instability or double-lung transplant in very sick patients, meticulous management of anticoagulation and perfusion flows is required to minimize inflammatory activation [113,114,115].

ECMO has become an important rescue strategy in severe PGD. If a patient develops life-threatening PGD, veno-venous ECMO allows the patient’s blood to be oxygenated externally, “resting” the transplanted lung [12]. ECMO support for a few days can allow the reperfusion injury to abate while avoiding additional ventilator-induced injury [116,117]. Clinical series have reported successful recovery of graft function in many PGD patients bridged through the acute phase on ECMO [118]. Thus, in an expert center, early deployment of ECMO for refractory PGD is considered a protective measure to prevent multi-organ failure. Overall, a combination of gradual reperfusion, protective ventilation, careful circulatory management, and timely supportive interventions form the cornerstone of PGD prevention and management in the perioperative setting [119,120].

### 3.5. LIRI in Donation After Circulatory Death

Donation after circulatory death (DCD) presents a distinct scenario for lung transplantation, with unique implications for IRI compared to donation after brain death (DBD) [121]. In controlled DCD, the agonal phase is the interval after withdrawal of life-sustaining therapy (WLST) until circulatory arrest (followed by a 2–5 min “no-touch” period before declaration of death) [122]. During this time, donors commonly exhibit intense spontaneous respiratory efforts/gasping with progressive hypoxemia and hypercapnic acidosis. These conditions drive patient self-inflicted lung injury (P-SILI), large negative intrathoracic pressure swings, “pendelluft”, and cyclic recruitment/derecruitment that promote edema and epithelial injury, thereby sensitizing the alveolar–capillary barrier to reperfusion [123,124,125]. Furthermore, they trigger hypoxic pulmonary vasoconstriction and acidosis-augmented increases in pulmonary vascular resistance, raising microvascular shear at first reflow [126,127]. In parallel, warm low-flow/no-flow metabolism accelerates ATP depletion and succinate accumulation, so that reperfusion rapidly generates mitochondrial ROS via reverse-electron transport at complex I, amplifying the endothelial injury that characterizes LIRI [24,87,128]. Hypoxia and hypercapnia also impair alveolar fluid clearance by down-regulating ENaC/Na, K-ATPase, further predisposing to reperfusion edema once circulation resumes [129,130,131]. These agonal-phase stresses explain why real-world tolerance is often shorter than the experimental “~60 min if oxygenated” benchmark, and why many programs target strict limits on functional warm ischemic time (e.g., from SBP < 50 mmHg or SpO_2_ < 70% to cold flush) rather than WLST-to-flush alone [132,133].

In controlled DCD (Maastricht category III, where withdrawal of life support is planned in a hospital setting), the agonal time can be anticipated and kept short. Indeed, it is a guiding principle that the agonal phase be <1 h to prevent irreversible lung injury [134]. By contrast, uncontrolled DCD (unexpected arrest) is more challenging due to indeterminate down-time; lungs from uncontrolled DCD (uDCD) donors are far less commonly used, or they may require ex vivo assessment before use [135]. Earlier data suggested that uncontrolled DCD lungs may be more prone to developing PGD; however, more recent data suggest that, when appropriately implemented, uDCD lung transplantation is feasible and safe [136].

Conversely, while DCD lungs suffer warm ischemic stress, they may avoid some of the injury associated with brain death. In DBD, a significant catecholamine storm often occurs at the time of brain herniation, causing severe hemodynamic swings and neurogenic pulmonary edema [137]. Additionally, brain death triggers systemic inflammatory responses that can prime donor organs with elevated cytokines and immune activation [68]. Studies have shown that some inflammatory markers are actually lower in DCD donor lungs compared to DBD [138]. Transcriptomic analyses similarly show that DBD lungs have upregulation of inflammatory pathways, due to a catecholamine surge and systemic “cytokine storm,” which primes the lung endothelium and leukocyte trafficking, thereby pre-activating inflammatory arms of IRI even before cold storage. DCD lungs, by contrast, show more activation of hypoxia and cell-death pathways due to the warm ischemia insult, on reperfusion, this state favors a mitochondrial ROS burst and sterile inflammation, rather than leukocyte-primed injury [63]. In practical terms, this means each donor type has a different profile of injury: DBD lungs are “pre-injured” by inflammation before ischemia, and DCD lungs are injured by lack of perfusion/oxygen during warm ischemia [138]. Both mechanisms can lead to PGD, via different routes. One notable difference is that inducible nitric oxide synthase (iNOS) signaling is more activated in marginal DBD lungs than in DCD lungs, suggesting that NO-related oxidative injury may be a feature of brain death-related lung damage [138]. Nevertheless, the key determinant in DCD lung outcomes is the duration of warm ischemia; longer durations correlate with more severe mitochondrial dysfunction and structural injury in donor organs [139,140,141]. Therefore, aggressive measures are taken to mitigate warm ischemia in DCD: as soon as the donor is pronounced dead, the transplant team will intubate/re-intubate and ventilate the donor, instill preservation solution into the lungs, and cool the organs as quickly as possible [68,134,142]. In some centers, normothermic regional perfusion (NRP) is used for DCD donors, using ECMO within the donor’s body to perfuse the organs with oxygenated blood before procurement [143]. NRP has been explored as a way to shorten the effective warm ischemic time and possibly recondition DCD lungs in situ, and there is evidence that it may reduce PGD, although data are still emerging [144,145]. Additionally, EVLP is playing a pivotal role in DCD lung transplantation. In a growing number of centers, lungs from both DBD and DCD donors are assessed on EVLP after procurement to ensure they meet functional criteria before implantation [146,147]. EVLP can also be used to treat donor lungs by removing excess fluid and delivering antioxidants or anti-inflammatory agents prior to transplant, thus acting as a bridge to improve DCD lung quality [20].

PGD remains a concern in DCD lung transplants, but recent data show that while DCD recipients may experience slightly higher rates of early PGD, the difference levels out within 72 h. Kashem et al. noted in their multicenter analysis of lung transplantation after DCD vs. DBD that at 1 h post-transplant the DCD group had more frequent severe PGD than the DBD group, but by 48–72 h there was no significant difference in PGD incidence between the two groups [148]. It appears that any initial damage in DCD lungs can often be overcome with supportive care, and the lung recovers similarly to a DBD lung. Importantly, when DCD protocols are properly followed (i.e., short agonal time, careful handling, use of EVLP when needed), the rates of successful transplantation are high [132,149,150].

## 4. Treatment Strategies

**Figure 2:**
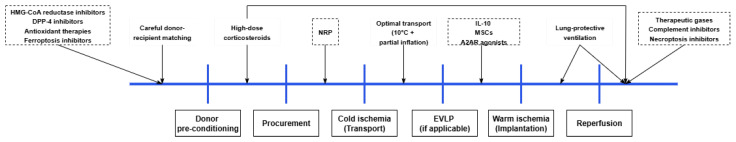
Overview of established and experimental treatment strategies within a lung transplant timeline. Each treatment strategy has been placed at its respective timing of (proposed) use. Dotted boxes indicate experimental/proposed strategies. A2AR, adenosine A_2A_ receptor; DPP-4, dipeptidyl peptidase 4; EVLP, ex vivo lung perfusion; HMG-CoA, 3-hydroxy-3-methylglutaryl coenzyme A; IL, interleukin; MSCs, mesenchymal stem cells; NRP, normothermic regional perfusion; NADPH, nicotinamide adenine dinucleotide phosphate; ROS, reactive oxygen species; TNF, tumor necrosis factor.

Due to the fact that LIRI involves a complex cascade with multiple overlapping pathways, a multi-faceted approach is necessary. Strategies include modifying surgical and perfusion techniques (to minimize ischemic time and reperfusion shocks), non-pharmacologic interventions (ventilatory strategies, extracorporeal support, positioning, conditioning), and pharmacologic or cellular therapies (antioxidants, anti-inflammatories, etc.). Below are some of the possible treatment strategies that have been proposed. The origin of the data regarding these treatment strategies vary from small-animal and large-animal models to EVLP studies using human donor lungs and clinical data (observational cohorts and randomized trials). Table 1 shows the mechanisms, evidence classes, net signals and translational status for each intervention category. Figure 2 shows a timeline overview of the different treatment modalities for LIRI, both established and experimental.

### 4.1. Lung-Protective Ventilation and Hypercapnia

Lung-protective ventilation is a key strategy in mitigating lung injury in the context of lung transplantation and other LIRI scenarios [106]. Mechanical ventilation can worsen pulmonary damage by inducing shear stress, volutrauma, and biotrauma, which in turn activate proinflammatory pathways such as nuclear factor kappa B (NF-κB) and mitogen-activated protein kinase, increase endothelial permeability, and promote cytokine release and neutrophil infiltration [96,106,151]. Evidence from ARDS literature and transplant cohorts supports the use of low tidal volumes (typically <6–8 mL/kg predicted body weight) combined with appropriate levels of PEEP to reduce these effects [12,106,152,153]. In a large retrospective study by Tague et al., donor-size–indexed low tidal volume ventilation after bilateral lung transplantation was associated with significantly lower rates of primary graft dysfunction (PGD) grade 3 and increased ventilator-free days [70]. Mechanistic insights from translational studies have shown that IRI activates sterile inflammation, damages the alveolar–capillary barrier, and leads to diffuse alveolar damage; hallmarks of PGD that can be attenuated through protective ventilator settings [15,154]. International survey data confirm that most transplant centers now routinely implement lung-protective ventilation strategies in the early post-reperfusion period, reflecting broad consensus on their clinical benefit [155]. These practices mirror findings from large ARDS trials, where limiting tidal volume and maintaining moderate PEEP consistently reduced inflammation, mechanical stress, and mortality [156,157,158]. Together, the preclinical rationale and clinical data make a compelling case for protective ventilation as a central component of supportive care during and after lung transplantation. Some experimental studies have suggested that a moderate hypercapnic acidosis (e.g., by reducing minute ventilation) can have protective effects. In isolated rat lungs, hypercapnic acidosis attenuated IRI, and in in vivo studies hypercapnia reduced lung injury by decreasing TNF-α levels, vascular leak, and apoptosis [159,160,161,162]. Hypercapnia appears to inhibit NF-κB activation and free radical generation, thereby blunting inflammation and cell death [12].

### 4.2. EVLP

In 2000, Steen et al. successfully performed the first lung transplantation using DCD lungs after evaluating the donor lungs using a novel technique called EVLP, which led to the development of the “Lund protocol” [163,164]. Subsequently, the Toronto group reported the results of a significant clinical trial using EVLP to evaluate the function of marginal donor lungs and DCD lungs, which laid the foundation for the “Toronto protocol” [20]. The group conducted 372 EVLP procedures between 2008 and 2017, increasing the annual lung transplantation rate by 70% during this period. EVLP has emerged in the last decade as a platform to rehabilitate and assess donor lungs before transplantation. Donor lungs are perfused and ventilated at normothermia in a specialized circuit for several hours. Clinically, EVLP allows marginal lungs to be evaluated and even treated prior to implantation [12]. Cypel et al. demonstrated that high-risk donor lungs treated with 4 h of EVLP had post-transplant outcomes comparable to standard lungs [165]. EVLP reduces the effective IRI burden by washing out DAMPs/cytokines linked to PGD, minimizing leukocyte/complement activation with acellular perfusate, and metabolically re-priming the graft under controlled flow/FiO_2_. These effects diminish succinate-driven mitochondrial ROS at reperfusion and has been associated with less warm-ischemia-related damage and lower PGD3 after transplant [87,91,166,167,168,169,170,171]. In a retrospective, single-center study of 538 lungs from the Toronto Lung transplant Program database, lungs that received EVLP recovered significantly faster from PGD grade 3 compared to lungs that did not receive EVLP [172]. Similarly, in a recent United Network for Organ Sharing database analysis of 10,342 patients that underwent lung transplantation between 2015 and 2023, EVLP was associated with decreased PGD grade 3 development [171]. These promising results of EVLP have led to the development of the Organ Care System (OCS) Lung™ (TranMedic, Andover, MA, USA), a portable EVLP platform that enables normothermic perfusion with donor blood and ventilation of the donor lungs during storage and transportation. In contrast to standard EVLP, this mobile system could potentially recondition donor lungs as soon as they are procured and (almost) completely eliminate cold ischemia during transport [173,174]. Very recently, the results of the EXPAND trial were published, a prospective, single-arm multicenter trial that assessed long-term clinical outcomes after use of OCS before transplantation of extended criteria donor lungs. The outcomes were compared to control cohorts from the same centers. The results showed similar 5-year overall survival rates. Furthermore, no significant differences were found between the EXPAND and control cohorts for the development of any grade of bronchiolitis obliterans syndrome [59].

EVLP can also serves as a delivery platform for therapies: drugs, gene therapy vectors, or cells can be administered ex vivo without risking systemic effects [91,175,176]. For example, experimental studies have shown that adenoviral IL-10 gene therapy given during EVLP in damaged human lungs led to improved lung function and reduced inflammation, making previously unusable lungs fit for transplant [177,178]. Likewise, EVLP studies in animal models show that adding treatments such as mesenchymal stem cells (MSCs), A2A adenosine receptor agonists, beta-2 agonists, or perfusate filtration can act on core LIRI pathways, lowering perfusate DAMP/cytokine burden and leukocyte activation, preserving endothelial/mitochondrial homeostasis, and improving alveolar fluid clearance, thereby reducing the reperfusion ROS/inflammation surge and improving early graft physiology [12,166]. EVLP is now routine in many major centers and has expanded the donor pool while also acting as a research bridge between bench and bedside for LIRI interventions [12,179].

### 4.3. Therapeutic Gases–NO, Carbon Monoxide, and Hydrogen

NO is a pulmonary vasodilator that can improve oxygenation and reduce PAP [180]. It has theoretical benefits in LIRI by improving perfusion and possibly modulating inflammation through cGMP signaling. By terminating lipid peroxidation chain reactions and S-nitrosation of mitochondrial complex I at the onset of reperfusion, it can theoretically dampen RET–mediated ROS generation [180,181]. Conversely, when superoxide levels are high, NO rapidly forms peroxynitrite (ONOO^−^), which amplifies oxidative/nitrative injury to lipids, proteins, and mitochondria; this helps explain why antioxidant benefits may not consistently translate at the bedside [182,183]. In practice, many transplant centers administer inhaled NO after reperfusion for severe PGD [10,12]. However, a double-blinded, placebo-controlled randomized trial in 84 lung transplant patients found no significant reduction in PGD incidence or outcomes with prophylactic inhaled NO [184]. Thus, inhaled NO is used as a supportive rescue therapy for hypoxemia, but evidence does not support it as a definitive preventive treatment for LIRI [185].

Low-dose CO exerts anti-inflammatory and anti-apoptotic effects via heme oxygenase-1 (HO-1) and stabilization of hypoxia-inducible factor (HIF-1α) [186,187]. In animal models of IRI, inhaling low concentrations of CO reduced IL-6 and TNF-α levels, decreased cell death, and improved oxygenation [188]. CO appears to induce a preconditioning-like state, upregulating cytoprotective pathways. A key mechanism is CO’s ability to modulate macrophage activation toward an anti-inflammatory phenotype and to suppress NF-κB [189,190]. Some early clinical studies in other organ transplants hint at safety of low-dose CO inhalation [191,192].

Hydrogen gas is a reactive free radical scavenger that can neutralize hydroxyl radicals [193]. It also activates the Nrf2 antioxidant response [194]. In multiple other organ IRI models, inhaled H_2_ or hydrogen-rich solutions reduced oxidative damage and improved outcomes [12]. In the lung, H_2_ has been shown to attenuate inflammatory cytokines and cell death in LIRI by boosting endogenous antioxidants [195,196]. One murine study found that combining inhaled H_2_ with NO provided additive protection against LIRI [196]. The main limitation is delivery: H_2_ is flammable, thus giving it during organ transport or to patients poses logistic challenges. However, hydrogen-rich saline can be administered intravenously or used as an organ flush [197]. In rat and LIRI models, hydrogen-enriched preservation solutions significantly reduced reperfusion injury [10,196].

### 4.4. Pharmacological Therapies

Several pharmacological therapies have been proposed as possible treatments or preventive measures for LIRI. Activation of complement is a key event in reperfusion injury [40]. Complement system activation increases the inflammatory reaction by producing anaphylatoxins such as C3a and C5a, resulting in an increased vascular permeability. In a multicenter cohort study, patients with increased plasma C5a levels at 6 h and 24 h post lung transplant were more likely to develop severe PGD and had higher mortality rates [39,40]. Furthermore, patients with PGD had higher bronchoalveolar lavage levels of complement activation fragments 24 h after lung transplantation [39].

HMG-CoA (3-hydroxy-3-methylglutaryl coenzyme A) reductase inhibitors (statins) have anti-inflammatory and endothelial-stabilizing properties beyond cholesterol lowering [198]. Observational studies indicate that lung transplant recipients on statin therapy have lower rates of PGD [199,200]. In a retrospective analysis (1999–2014), PGD occurred in 34.8% of patients on perioperative statins vs. 57.9% of those not on statins [199]. After propensity adjustment, statin use was independently associated with reduced PGD risk (OR 0.4). Some centers now consider continuing or starting statins in the peri-transplant period as an adjunct protective strategy [199,200].

High-dose corticosteroids are often given to the donor before organ procurement and to the recipient before reperfusion, primarily to attenuate immune activation and hyperacute rejection [201]. While not studied in randomized trials specifically for IRI prevention, steroids likely attenuate some early cytokine surges in LIRI (TNF, IL-1, etc.) [202]. Almost all lung transplant protocols include steroids (partly for anti-rejection), so their effect is entangled with standard care [203]. Experimental data in animals support that steroids reduce LIRI edema and inflammation, but careful timing and dosing are needed to balance risks such as infection and impaired healing [204,205].

Because mitochondrial ROS bursts, bioenergetic collapse, and defective mitophagy are central in LIRI, several mitochondria-directed strategies are being pursued. Mitochondria-targeted antioxidants and peptides, such as elamipretide (SS-31), which binds cardiolipin to stabilize cristae, and mitochondrial quinones like MitoQ, aim to reduce electron leak and limit mitochondrial ROS. Although most efficacy data come from heart and kidney IRI, the mechanisms are directly relevant to LIRI and EVLP may allow organ-specific delivery [206,207,208]. Complementary “metabolic re-wiring” at reperfusion seeks to blunt reverse electron transport–driven ROS by modulating succinate dehydrogenase activity (e.g., malonate or dimethyl-malonate prodrugs) [128,209]. Restoring mitophagy and mitochondrial quality control via Sirtuin1 (SIRT1)–PTEN-induced kinase 1 (PINK1)/Parkin signaling, illustrated by adiponectin-mediated rescue in diabetic lung-transplant models, can preserve mitochondrial integrity and reduce injury [210]. An orthogonal approach is mitochondrial transfer or transplantation, either through endogenous MSC-to-epithelium donation or exogenous delivery, which has improved lung mechanics, ATP content, and inflammation in preclinical lung-injury studies [46,211,212]. Collectively, these modalities are biologically compelling yet supported by limited lung-specific clinical data.

Metformin has emerged as a possible treatment candidate in LIRI. In a type-2 diabetic rat LTx model, metformin improved graft function and reduced necroptosis by activating adenosine monophosphate-activated protein kinase (AMPK); its benefits were reversed by an AMPK inhibitor, supporting on-target activity. Independent groups have replicated protection in LTx IRI models and extended benefits to CLAD in rats [213,214]. Mechanistically, metformin’s complex-I modulation dampens mitochondrial ROS and engages AMPK-dependent cytoprotective pathways that intersect with mitophagy and endothelial barrier preservation, pathways already implicated in diabetic LIRI. A 2024–2025 cross-organ IRI literature synthesis positions metformin as a plausible peri-reperfusion adjunct, though no randomized human LTx trials have reported to date [215,216].

### 4.5. Anti-Inflammatory and Immunomodulatory Approaches

Activation of A2A receptors on immune cells has broad anti-inflammatory effects (increasing cAMP and inhibiting neutrophil/macrophage activation) [217,218]. In animal models of LIRI, selective A2A agonists given during EVLP improved lung compliance and reduced pro-inflammatory cytokines and neutrophil infiltration [219,220,221]. These promising results have led to a recent blinded multicenter randomized controlled trial of regadenoson, an A2A agonist, in lung transplant patients, recently showed a trend towards reducing IRI [222].

IL-10 is an anti-inflammatory cytokine that suppresses production of TNF-α, IL-1β, and other harmful mediators [223]. Gene therapy to overexpress IL-10 in donor lungs via EVLP has shown promising results [12]. In large animal models and human lungs deemed unsuitable for transplant, delivering IL-10 with an adenoviral vector during EVLP significantly attenuated subsequent reperfusion injury and even improved 30-day graft survival post-transplant [224,225]. IL-10 gene therapy effectively “preconditions” the lung by attenuating the inflammatory cascade upon reperfusion. To mitigate risks, the ex vivo delivery of the gene therapy confines it to the graft [179].

Dipeptidyl peptidase-4 (DPP-4/CD26) is a cell-surface serine protease highly expressed in the lung on capillary endothelium and alveolar cells [226,227]. It inactivates several cytoprotective peptides: GLP-1, vasoactive intestinal peptide (VIP), and stromal cell–derived factor-1α (SDF-1α) [17]. Pharmacologic DPP-4 inhibition preserves these substrates and exerts anti-inflammatory, antioxidant, and endothelial-protective effects [17,228,229,230]. Mechanistically, VIP limits NF-κB activation, reduces caspase activity, and suppresses pro-inflammatory cytokines while promoting broncho- and vasodilation; DPP-4 inhibition increases VIP-positive macrophages after reperfusion [17,231,232,233,234]. SDF-1α recruits bone-marrow progenitors and activates cell-survival signaling via CXCR4; DPP-4 inhibition raises intact SDF-1α and enhances progenitor homing [229,235]. GLP-1 receptor activation elevates cAMP, downregulates NADPH oxidase (reducing superoxide), and induces endogenous antioxidant enzymes; in endotoxemia models, DPP-4 inhibitors reduce vascular oxidative stress, NOX2 expression, inducible NOS, NF-κB activation, and inflammatory cytokine release while upregulating SOD, GPx, and thioredoxin [236,237,238,239,240,241,242].

Preclinical lung IRI and transplant studies corroborate these mechanisms. In ex vivo rat lung perfusion, irreversible DPP-4 inhibition improved oxygenation and lowered pulmonary artery pressures; in vivo, pretreatment improved compliance, reduced edema and neutrophil infiltration, and lessened histologic injury, with greater VIP staining [228]. In a mouse orthotopic transplant with 18 h cold ischemia, DPP-4 inhibition improved post-reperfusion function, reduced macrophage/neutrophil activation, and attenuated acute rejection. Donor-lung preconditioning with vildagliptin during cold storage decreased macrophage/neutrophil influx and ICAM-1 expression, improved oxygenation, and yielded better structure and function up to 14 days, accompanied by higher SDF-1α, more IL-10, and M2 macrophage polarization; CD26-knockout recipients showed similar protection, implicating DPP-4 activity directly [229].

Human data are limited to a retrospective diabetic-recipient cohort, where DPP-4 inhibitor use associated with higher 5-year survival and freedom from CLAD, and explanted tissues from non–DPP-4i patients showed higher CD26 expression [26]. While encouraging, these findings require confirmation in prospective trials to define dosing, timing (donor, EVLP, or recipient), and patient selection for preventing LIRI and possibly reducing downstream CLAD.

Bone marrow-derived MSCs possess immunomodulatory and pro-repair properties. They can release anti-inflammatory cytokines, growth factors, and microvesicles that reduce endothelial permeability and promote tissue repair [10]. In mouse lung transplant models, MSC infusion protected lungs from IRI, reducing inflammatory cell infiltration and apoptosis [10]. MSC-derived extracellular vesicles (EVs), nano-sized vesicles enriched in miRNAs, proteins and lipids, recapitulate much of the parent cells’ immunomodulatory and reparative effects while avoiding concerns about persistence, embolism, or ectopic differentiation. These MSC-derived EVs have similarly shown potential to attenuate lung inflammation in IRI by reducing neutrophil influx and cytokine release, preserving alveolar–capillary barrier function, and improve oxygenation. Mechanisms include transfer of regulatory miRNAs (e.g., miR-21-5p, miR-146a) to alveolar macrophages and epithelial cells, promotion of epithelial repair programs, and enhancement of alveolar fluid clearance [195,197,243,244,245]. Several studies have indicated that MSC therapy protects the alveolar-epithelial barrier during ALI, likely due to improved permeability, alveolar fluid clearance, and oxygenation [12,246]. Safety of allogeneic MSCs has been shown in a Phase 2a trial for ARDS where no infusion-related toxicity was demonstrated [247]. Consequently, early-phase trials of MSC therapy for lung transplant patients (to prevent PGD or treat chronic rejection) are in development [246]. Two delivery avenues are especially promising for translation. First, EVLP provides a controllable window to administer EVs (or engineered EVs) directly to donor lungs before implantation. Contemporary reviews highlight EVLP as a practical bridge for EV/MSC products in LTx, with dosing and manufacturing standardization as the next hurdles [179,243,248]. Second, regional airway delivery is feasible: nebulized, miR-146a–engineered human umbilical cord MSC-EVs attenuated LIRI and improved graft oxygenation in an orthotopic rat LTx model by suppressing NLRP3 inflammasome signaling. Earlier work also showed anti-inflammatory activity of human MSC-EVs in reperfusion models [249].

During LIRI, endogenous danger signals like HMGB1 and extracellular ribonucleic acid (RNA) trigger toll-like receptors (TLRs) and inflammasomes. Experimental agents that neutralize HMGB1 or block TLR4 signaling (e.g., TLR4 antagonist eritoran) have shown reductions in LIRI in animal studies [10]. Similarly, inhibiting the NOD-like receptor family pyrin domain containing 3 (NLRP3) inflammasome or caspase-1 to reduce pyroptosis, a form of inflammatory cell death, is being explored. For instance, one study found that a glucagon-like peptide-1 (GLP-1) receptor agonist added during EVLP modulated pyroptosis pathways and improved lung graft function [236].

An often under-recognized contributor to LIRI is microthrombosis in the pulmonary microvasculature. Donor lungs, especially from DCD cases, can contain fibrin clots that impede reperfusion and prolong ischemia in parts of the lung [250]. Fibrinolytics like tissue plasminogen activator (tPA) or plasmin given to the donor lung have been tested to dissolve these clots [251,252]. Notably, administering plasmin during EVLP effectively cleared microthrombi and led to improved post-transplant lung function in recent studies [10]. Some EVLP protocols routinely include fibrinolytic treatment, particularly for DCD lungs where intravascular coagulation is a concern [10,253].

Another possible therapy includes inhibiting necroptosis, a programmed necrosis driven by receptor-interacting serine/threonine-protein kinases 1 and 3 (RIPK1/RIPK3) and mixed lineage kinase domain like pseudokinase (MLKL), contributing to endothelial and epithelial death in IRI [254]. Necrostatin-1 (Nec-1), a RIPK1 inhibitor, has shown efficacy in a mouse hilar clamp model of LIRI [255]. Nec-1 treatment significantly reduced lung injury scores, lowered pro-inflammatory cytokines, and decreased necroptotic markers in lung tissue. In vitro, Nec-1 also protected pulmonary cells from cold hypoxia-reoxygenation injury by preventing necrosome (RIPK1/RIPK3 complex) formation. Interestingly, Nec-1 not only blocked necroptosis but also reduced caspase-dependent apoptosis of epithelial cells, and it promoted apoptosis of neutrophils, thereby accelerating resolution of inflammation. By inhibiting both necroptosis and apoptosis, and encouraging clearance of neutrophils, Nec-1 provided multi-faceted protection. The authors concluded Nec-1 could be a promising therapy to prevent PGD [255]. Similarly, necrosulfonamide (an MLKL inhibitor) has shown protective effects in a rat LIRI model by blocking necroptosis of airway cells [256]. These findings underscore the therapeutic potential of targeting regulated necrosis in LIRI.

### 4.6. Antioxidant Therapies

Classic antioxidants like N-acetylcysteine (NAC), vitamin C, and superoxide dismutase mimetics have been studied in various IRI models [257,258,259]. NAC, a glutathione precursor, has been shown in some animal studies to reduce LIRI edema and malondialdehyde (lipid peroxidation) levels, although high doses are needed [259,260]. While antioxidants are intuitively beneficial, human data are limited.

Beyond inhaled NO as a vasodilator, strategies to enhance endogenous NO signaling are considered cytoprotective. For example, nitrite (NO_2_^−^) can be enzymatically reduced to NO during ischemia, acting as an oxygen-independent NO donor. In some organ IRI models, nitrite infusion before reperfusion improved microvascular flow and reduced ROS due to the fact that NO can outcompete superoxide to form peroxynitrite instead of hydroxyl radical formation [261,262]. Soluble guanylate cyclase activators, which amplify NO signaling, are another avenue; however, their role in LIRI is speculative [263]. Balancing NO is complex because excess inducible NOS during reperfusion can be harmful [10]. Antioxidant cocktails have not yet conclusively improved clinical outcomes in lung transplant or ARDS, possibly because timing and delivery to cells are challenging [264]. Nevertheless, managing oxidative stress remains a cornerstone concept, and these agents could be part of a multimodal regimen in the future.

Another possible avenue is targeting ferroptosis, iron-dependent lipid peroxidation-driven cell death [10]. Injured lungs often show signs of ferroptosis: iron accumulation, depleted glutathione, lipid peroxides, and several methods of targeting this pathway have been proposed in recent years [265]. Maresin-1 (MaR1), an endogenous pro-resolving lipid mediator, was recently shown to protect mouse lung transplants by inhibiting ferroptosis [10]. In a 2025 study, MaR1 given to mice undergoing lung transplantation preserved glutathione levels, prevented lipid peroxidation, and reduced ultrastructural mitochondrial damage in grafts [266]. Mechanistically, MaR1 activated Protein Kinase A and the Hippo-YAP pathway, which in turn reduced ferroptosis signaling. The authors concluded that MaR1 effectively attenuated LIRI and suggested it as a basis for organ-protective therapy. Beyond MaR1, classical iron chelators like deferoxamine or lipophilic antioxidants such as liproxstatin-1 could theoretically be applied to donor lungs to curb ferroptotic injury [267]. This area is still emerging, but targeting ferroptosis is promising given its central role in IR-induced cell death [10].

## 5. Conclusions

LIRI remains an important cause of morbidity and mortality in thoracic surgery, particularly in the context of lung transplantation, where it is the principal driver of PGD and a major contributor to the development of CLAD. Despite advances in surgical technique, organ preservation, and perioperative care, the incidence and impact of LIRI remain substantial, particularly with increasing reliance on extended-criteria donors and DCD lungs.

Over the past decade, considerable progress has been made in deciphering the complex pathobiology of LIRI, including the central roles of oxidative stress, endothelial dysfunction, inflammatory cell recruitment, and disruption of the alveolar–capillary barrier. Experimental models have further elucidated mechanisms such as endothelial glycocalyx degradation, mitochondrial injury, and regulated cell death pathways, all of which converge to exacerbate pulmonary edema, gas exchange impairment, and long-term allograft dysfunction.

Clinically, the evolution of lung-protective ventilation strategies, controlled reperfusion protocols, and the application of EVLP has improved early postoperative outcomes and expanded the donor pool. These advances reflect the growing recognition that a multimodal and phase-specific approach to LIRI prevention, beginning with donor management and extending through procurement, implantation, and postoperative care, is essential.

While different pharmacologic interventions have been proposed, including antioxidants, complement inhibitors, vasodilators, and immunomodulatory agents, few have translated successfully into routine clinical use. However, the growing understanding of cellular and molecular targets continues to inform the development of novel therapies. These include strategies to preserve the endothelial barrier, modulate innate immune activation, and inhibit key inflammatory mediators. Emerging approaches such as mesenchymal stem cell therapies, cytokine modulation, and inhibition of regulated necrosis (e.g., necroptosis, ferroptosis) hold particular promise but require further clinical validation.

LIRI spans heterogeneous donor and recipient states (DBD vs. DCD, ischemic times, size mismatch, pulmonary hypertension, ECMO use), making effect sizes small and context-dependent. Timing and route of delivery (intra-donor, EVLP, intra-op reperfusion, early post-op) are critical yet inconsistently standardized. Surrogate endpoints vary (e.g., PGD grade vs. biomarker panels), and negative or neutral trials of intuitive therapies (e.g., prophylactic inhaled NO) highlight the need for biomarker-guided enrichment and platform trials. Manufacturing and regulatory issues also slow cellular/EV products. We outline these issues to guide realistic translation of promising preclinical findings.

The future management of LIRI will depend on the integration of mechanistic insights with real-time biomarkers for injury prediction, early detection, and therapeutic monitoring. Translational and clinical research need to bridge the gap between bench and bedside, ideally through multicenter trials and standardized outcome measures.

## Figures and Tables

**Table 1:** Evidence map of interventions targeting lung ischemia–reperfusion injury (LIRI)/primary graft dysfunction (PGD) in lung transplantation.

Intervention	Proposed Mechanism(s)	Evidence Classes	Net Signal	Translational Status
LPV ± permissive hypercapnia	- ↓ Volutrauma/atelectrauma - Hypercapnia → ↓ NF-κB signaling and ROS injury	- Small animal ✓ - Human observational ✓	- LPV favorable - Hypercapnia preclinical favorable	- LPV standard of care - Hypercapnia investigational
Controlled/gradual reperfusion at implantation	Pressure/flow-limited reperfusion → ↓ endothelial shear/ROS burst	- Small animal ✓ - Large animal ✓ - Human observational ✓	Favorable	Standard of care
EVLP	- Normothermic assessment and reconditioning - Reduces ischemic time - Delivery vehicle for therapies	- Large animal ✓ - Human observational ✓ - RCT non-inferior vs. conventional preservation	Favorable	Established adjunct in many centers
iNO	- Selective pulmonary vasodilation - Improves V/Q matching - Theoretical anti-inflammatory effects	- Human RCT	- Neutral for PGD prevention - Useful as rescue/support	Supportive therapy
iCO	- Cytoprotective gas - Anti-inflammatory/anti-apoptotic via HO-1 and mito-signaling	- Small animal ✓ - Large animal ✓	Favorable (preclinical)	Investigational
iH_2_	- Scavenges •OH/ONOO^−^ - Activates Nrf2/HO-1 - Anti-apoptotic	- Large animal ✓ - EVLP (DCD) ✓	Favorable (preclinical/EVLP)	- Investigational - EVLP add-on candidate
Statins (pre-LTx exposure)	Pleiotropic anti-inflammatory and endothelial stabilizing	- Human observational ✓	Mixed/neutral	Not recommended specifically for PGD prevention
High-dose corticosteroids (peri-operative)	General anti-inflammatory part of standard immunosuppression	- Human observational ✓	Neutral for PGD prevention	Standard immunosuppression; not targeted PGD therapy
Mitochondria targeted agents	- Preserve mitochondrial integrity - Reduce mPTP opening - Reverse-electron-transport ROS	- Small animal ✓ - Large animal ✓ - EVLP model ✓	Favorable (preclinical)	Investigational
Metformin	- Activates AMPK - Dampens inflammation/oxidative stress - May limit necroptosis	- Small animal ✓	Favorable (preclinical)	Investigational (repurposing candidate)
DPP-4i	- Preserve VIP/chemokine tone - Anti-inflammatory/anti-apoptotic	- Small animal ✓ - Large animal ✓ - Human observational ✓	Favorable signal	Investigational (early clinical signal)
A_2_A RA	- Immune-cell deactivation - Limits neutrophil-endothelium interactions	- Small animal ✓ - EVLP ✓ - RCT ✓	Biomarker signal; clinical efficacy unproven	Early-phase clinical; more trials needed
IL-10 therapy	- Potent anti-inflammatory cytokine - Shifts macrophage/T-cell responses	- Large animal ✓ - EVLP model ✓	Favorable (preclinical)	Investigational (EVLP delivery under refinement)
MSCs	- Paracrine immunomodulation - Antioxidant & endothelial protection	- Small animal ✓ - Large animal ✓ - EVLP model ✓	Favorable (preclinical); early human safety	Investigational/early clinical
“Classic” antioxidants	- Direct ROS scavenging - Glutathione repletion	- Small animal ✓ - EVLP model ✓	Inconsistent; no proven clinical benefit for PGD	Stalled—insufficient evidence for routine use
TLR pathway antagonists	Dampen innate immune activation to DAMPs	- Small animal ✓	Favorable (preclinical)	Investigational
Fibrinolytics	- Dissolve donor pulmonary thrombi - Improve perfusion/oxygenation	- EVLP model ✓	Favorable in selected EVLP cases	Selective EVLP tool; not PGD-directed therapy
Necroptosis inhibitors	- Block RIPK1/RIPK3/MLKL-mediated regulated necrosis	- Small animal ✓	Favorable (preclinical)	Investigational
Ferroptosis inhibitors	- Limit iron-dependent lipid peroxidation and cell death	- Small animal ✓	Favorable (preclinical)	Investigational

AMPK, AMP-activated protein kinase; A_2_A RA, adenosine A_2_A receptor agonist; DAMPs, damage-associated molecular patterns; DCD, donation after circulatory death; DPP-4i, dipeptidyl peptidase-4 inhibitor(s); EVLP, ex vivo lung perfusion; HO-1, heme oxygenase-1; iCO, inhaled carbon monoxide; iH2, inhaled hydrogen; IL-10, interleukin-10; iNO, inhaled nitric oxide; LIRI, lung ischemia–reperfusion injury; LPV, lung-protective ventilation; LTx, lung transplantation; MLKL, mixed lineage kinase domain-like protein; mPTP, mitochondrial permeability transition pore; MSCs, mesenchymal stromal cells; NF-κB, nuclear factor kappa-light-chain-enhancer of activated B cells; Nrf2, nuclear factor erythroid 2-related factor 2; ONOO-, peroxynitrite; PGD, primary graft dysfunction; RCT, randomized controlled trial; RIPK1, receptor-interacting serine/threonine-protein kinase 1; RIPK3, receptor-interacting serine/threonine-protein kinase 3; ROS, reactive oxygen species; TLR, Toll-like receptor; V/Q, ventilation/perfusion; VIP, vasoactive intestinal peptide; •OH, hydroxyl radical.

## Data Availability

No new data were created or analyzed in this study. Data sharing is not applicable to this article.

## Abbreviations

The following abbreviations are used in this manuscript:

ALI	Acute Lung Injury
ARDS	Acute Respiratory Distress Syndrome
ATP	Adenosine Triphosphate
BAL	Bronchoalveolar lavage
BMI	Body Mass Index
cAMP	Cyclic Adenosine Monophosphate
CD26	Cluster of Differentiation 26
cGMP	Cyclic Guanosine Monophosphate
CLAD	Chronic Lung Allograft Dysfunction
CO	Carbon Monoxide
COPD	Chronic Obstructive Pulmonary Disease
COR	Controlled Oxygenated Rewarming
CPB	Cardiopulmonary Bypass
CVP	Central Venous Pressure
CXCL	C-X-C Ligand
DAMP	Damage-associated Molecular Patterns
DBD	Donation after Brain Death
DCD	Donation after Circulatory Death
DNA	Deoxyribonucleic Acid
DPP-4	Dipeptidyl Peptidase-4
ECMO	Extracorporeal Membrane Oxygenation
EV	Extracellular Vesicles
EVLP	Ex Vivo Lung Perfusion
FiO_2_	Fraction of Inspired Oxygen
GLP	Glucagon-like Peptide
GM-CSF	Granulocyte-Macrophage Colony-Stimulating Factor
HIF	Hypoxia-inducible Factor
HMG-CoA	3-Hydroxy-3-Methylglutaryl Coenzyme A
HMGB1	High Mobility Group Box 1
HOPE	Hypothermic Oxygenated Perfusion
ICU	Intensive Care Unit
IL	Interleukin
iNOS	Inducible Nitric Oxide Synthase
IR	Ischemia–Reperfusion
IRI	Ischemia–Reperfusion Injury
IV	Intravenous
KATP	ATP-Sensitive Potassium Channel
LIRI	Lung Ischemia–Reperfusion Injury
MaR1	Maresin-1
MLKL	Mixed Lineage Kinase Domain Like Pseudokinase
MOF	Multiple Organ Failure
MSC	Mesenchymal Stem Cell
NAC	N-acetylcysteine
NADPH	Nicotinamide Adenine Dinucleotide Phosphate
NET	Neutrophil Extracellular Traps
NF-κB	Nuclear Factor Kappa B
NLRP3	NOD-like Receptor Family Pyrin Domain Containing 3
NMP	Normothermic Machine Perfusion
NO	Nitric Oxide
NOS	Nitric Oxide Synthase
NOX	NADPH Oxidase 2
NRP	Normothermic Regional Perfusion
OCS	Organ Care System
OLV	One-lung Ventilation
PAP	Pulmonary Artery Pressure
PECAM-1	Platelet Endothelial Cell Adhesion Molecule
PEEP	Positive End-Expiratory Pressure
PGD	Primary Graft Dysfunction
PI3K-Akt	Phosphoinositide 3-Kinase–Akt pathway
PINK1	PTEN-induced kinase 1
RANTES	Regulated upon Activation, Normal T cell Expressed and Secreted
RIC	Remote Ischemic Conditioning
RIPK1/3	Receptor-interacting Serine/Threonine-Protein Kinases 1/3
RNA	Ribonucleic Acid
ROS	Reactive Oxygen Species
SDF	Stromal-cell Derived Factor
SIRT1	Sirtuin 1
TLR	Toll-like Receptor
TNF	Tumor Necrosis Factor
tPA	Tissue Plasminogen Activator
uDCD	Uncontrolled Donation after Circulatory Death
VEGFR2	Vascular Endothelial Growth Factor Receptor 2
VIP	Vasoactive Intestinal Peptide
V/Q	Ventilation/Perfusion
XO	Xanthine Oxidase
